# A cancer-type-aware framework for robust multimodal survival prediction under missing modalities

**DOI:** 10.1093/bib/bbag124

**Published:** 2026-03-23

**Authors:** Yiran Song, Zaifu Zhan, Feng Xie, Nian Wang, Yifan Peng, Rui Zhang, Mingquan Lin

**Affiliations:** Division of Computational Health Sciences, University of Minnesota, Mayo D528, 420 Delaware St SE, Minneapolis, MN 55455, United States; Division of Computational Health Sciences, University of Minnesota, Mayo D528, 420 Delaware St SE, Minneapolis, MN 55455, United States; Department of Electrical and Computer Engineering, University of Minnesota, Minneapolis, MN 55455, United States; Division of Computational Health Sciences, University of Minnesota, Mayo D528, 420 Delaware St SE, Minneapolis, MN 55455, United States; Advanced Imaging Research Center, Department of Biomedical Engineering, Peter O'Donnell Jr. Brain Institute, 2201 Inwood Road, NE3.218, Dallas, TX 75390, United States; Department of Population Health Sciences, Weill Cornell Medicine, New York, NY 10065, United States; Division of Computational Health Sciences, University of Minnesota, Mayo D528, 420 Delaware St SE, Minneapolis, MN 55455, United States; Division of Computational Health Sciences, University of Minnesota, Mayo D528, 420 Delaware St SE, Minneapolis, MN 55455, United States

**Keywords:** multimodal learning, cancer prognosis, missing data, clinical AI, deep learning, survival analysis, cross-institutional validation, cancer heterogeneity

## Abstract

Despite advances in multimodal cancer prognosis, robust performance in practical settings remains hindered by three critical barriers: ubiquitous data incompleteness, failure to model cancer-specific biology, and cross-institutional instability. We address these practical challenges through a cancer-type-aware framework that uniquely combines adaptive gated fusion for missing modalities, hybrid architecture for cancer heterogeneity, and demonstrated cross-institutional robustness. By establishing histopathology as the universally available anchor modality while adaptively incorporating RNA expression and clinical text through gated fusion, our framework maintains robust performance under realistic data constraints. Evaluation across 10 The Cancer Genome Atlas cancer types demonstrated superior performance (C-indices 0.578–0.778; mean 0.670 $\pm $ 0.066), with state-of-the-art results in six cancer types. The framework maintained predictive performance under missing data scenarios, with C-indices ranging from 0.621 to 0.627 for missing RNA and from 0.568 to 0.606 for missing clinical text. Cross-institutional validation across 12–38 centers demonstrated robust cross-institutional performance (standard deviations <0.040 in eight of 10 cancer types). This methodological framework addresses key technical prerequisites—handling missing data, modeling cancer heterogeneity, and ensuring cross-institutional stability—for multimodal survival prediction, providing computational foundations necessary for future prospective clinical validation.

## Introduction

Cancer prognosis prediction is fundamental to precision oncology, guiding treatment decisions and improving patient outcomes for nearly 19 million new cases diagnosed annually worldwide [1, 2]. Accurate survival prediction enables personalized treatment planning, risk stratification, and informed prognostic assessment across diverse tumor types. Yet, traditional staging systems (e.g. TNM classification) capture only coarse anatomical features and fail to represent the molecular and histological heterogeneity that strongly influences patient survival, motivating the integration of richer multimodal data sources in modern prognostic assessment. Clinicians have traditionally integrated diverse multimodal data sources for comprehensive prognostic assessment [3], with histopathological images serving as the gold standard for diagnosis [4]. Gene expression profiles reveal molecular dynamics and therapeutic targets [5, 6], while clinical records capture treatment history and patient characteristics [7–9]. Although this human-centric integration is vital, the massive scale and complexity of these data sources make manual synthesis increasingly difficult, necessitating the development of automated prediction systems. Yet, creating these systems presents significant computational challenges, as they must effectively fuse disparate data types into a cohesive prognostic model.

To address these complexities, recent multimodal approaches have shown promise by integrating histopathological, genomic, and clinical data [10–16]. However, their clinical translation remains constrained by three fundamental technical challenges commonly encountered in real-world biomedical data:

**(1) Incomplete multimodal data**: In practice, multimodal datasets are often incomplete due to cost constraints (RNA sequencing: $1000–3000 per patient), institutional protocols, and technical limitations [17]. Most existing models require all modalities to be present at inference time, rendering them ineffective when auxiliary modalities are unavailable. Generative imputation approaches [18–22] and disentanglement-based architectures [23–27] attempt to address missing data but rely on strong distributional assumptions that degrade when test-time missing patterns differ from training distributions. Recent methods such as M3Surv [28] and LDCVAE [29] provide partial solutions, yet none offer sufficient flexibility to accommodate arbitrary missing modalities commonly encountered in computational oncology datasets, limiting robustness under realistic conditions.

**(2) Cancer commonality-heterogeneity balance:** Existing methods typically adopt either independent training for each cancer type or unified training across all cancers, but neither strategy optimally balances shared prognostic patterns and cancer-specific characteristics. Independent training for cancer-specific models [30–32] ignores cross-cancer commonalities and suffers from limited sample sizes, whereas unified training in pan-cancer models overlooks tumor-specific molecular signatures crucial for accurate prediction [33]. As a result, these methods struggle to leverage both pan-cancer biological principles and cancer-specific biomarkers, failing to capture the full spectrum of biological heterogeneity essential for robust prognosis.

**(3) Cross-institutional stability**: Many models exhibit substantial performance variability across institutional settings and data distributions (standard deviations <0.040 in 8 of 10 cancer types), indicating limited cross-site robustness when applied to data from institutions with varying protocols, imaging equipment, and patient populations [34]. This instability arises from multiple sources: variations in imaging protocols (different scanners, staining procedures), documentation styles (structured versus narrative clinical notes), and patient population characteristics (demographic and clinical heterogeneity). Models trained on single-institution data often overfit to institution-specific patterns, failing to capture robust prognostic signals that generalize across diverse data collection environments. Addressing this challenge requires explicit architectural design for cross-institutional robustness rather than relying solely on data augmentation or *post hoc* calibration.

Motivated by these challenges, we introduce a cancer-type-aware multimodal survival prediction framework that establishes histopathology as a universally available anchor modality and adaptively incorporates RNA expression and clinical text through a gated fusion mechanism capable of handling arbitrary missing-modality patterns. A hybrid architecture combining shared multimodal encoders with cancer-type-specific prediction heads enables the model to capture both cross-cancer prognostic structure and tumor-specific signatures. By designing directly around practical constraints—missing modalities, cancer heterogeneity, and institutional variability—the proposed framework provides a methodological solution addressing key technical challenges in multimodal survival prediction. Our approach achieves performance consistent with established computational benchmarks (mean C-index: 0.670$\pm $0.066) with demonstrated robustness to missing data and strong cross-institutional stability (standard deviation <0.040), providing computational foundations that address fundamental technical prerequisites for future validation in diverse institutional settings.

## Methods

### Dataset and experimental design

#### Data selection and cohort characteristics

We processed three modalities from The Cancer Genome Atlas (TCGA) data through standardized pipelines to ensure consistent feature representations across all cancer types. From the comprehensive TCGA database spanning 32 cancer types [35], we selected 10 cancer types with sufficient sample sizes ($>150$ patients) and complete multimodal data availability for robust evaluation: endometrial carcinoma of the uterine corpus (UCEC), lung adenocarcinoma (LUAD), lower grade glioma (LGG), breast invasive carcinoma (BRCA), bladder urothelial carcinoma (BLCA), pancreatic adenocarcinoma (PAAD), colon adenocarcinoma (COAD), rectum adenocarcinoma (READ), kidney renal clear cell carcinoma (KIRC), and glioblastoma multiforme (GBM). Each patient was characterized by survival outcomes measured from diagnosis to death or last follow-up, with survival times discretized into four bins for model training. Patients with survival times exceeding the maximum observed time within each cancer type were censored appropriately for survival analysis.

#### Data partitioning strategy

We employed multiple evaluation strategies to assess model robustness and generalizability, addressing critical technical challenges including institutional performance variability [34] and the need for tumor-specific modeling approaches [33].

##### Primary evaluation (4:2:4 split)

Our primary evaluation employed a 4:2:4 train-validation-test split strategy, deliberately designed to reflect realistic data constraints commonly encountered in computational oncology settings. This allocation strategy differs from conventional 7:1:2 splits in several important ways:

1. **Limited training data availability**: In practice, institutions often have limited access to complete multimodal datasets due to the high cost of RNA sequencing ($1000–3000 per patient), varying institutional protocols, and technical limitations [17]. The 40% training allocation simulates scenarios where computational models must be developed with constrained data resources.
2. **Robust test set evaluation**: Rigorous methodological evaluation requires assessment on substantial patient cohorts to ensure model reliability. The 40% test allocation (versus 20% in conventional splits) provides more statistical power to assess generalization performance and detect potential failures in cross-validation scenarios.
3. **Model stability assessment**: By intentionally limiting training data, this split strategy tests whether the model can maintain stable performance under data scarcity—a critical requirement for application across institutions with varying data collection capabilities.

##### Missing modality assessment

Robustness was evaluated by systematically excluding RNA and clinical text modalities for 20%, 50%, and 80% of patients during evaluation while consistently retaining histopathological features as the anchor modality [4]. This systematic evaluation reflects realistic scenarios where complete multimodal data are often unavailable due to cost constraints, institutional protocols, or technical limitations [17], while acknowledging that histopathological images serve as the diagnostic gold standard [4].

Importantly, these missing modality scenarios were **randomly generated during evaluation only**—the model was trained with structured modality dropout but did not see the specific test-time missing patterns. This evaluation protocol tests the model’s ability to **generalize to arbitrary missing-data scenarios not encountered during training**, a critical requirement for robustness where missing-data patterns vary unpredictably across institutions and individual patients.

##### Data split sensitivity analysis

We conducted a comparative analysis between 4:2:4 versus conventional 7:1:2 partitioning strategies to assess model stability under varying training data availability. This analysis demonstrates the framework’s stability across institutions with different data collection capabilities and addresses concerns about reproducibility and generalizability essential for cross-institutional applications [34].

#### Ethical considerations and data privacy

All analyses were conducted using publicly available TCGA data with appropriate institutional review board approvals from the original data collection [35]. Patient identifiers were de-identified in accordance with HIPAA guidelines and TCGA consortium standards. The framework design incorporates privacy-preserving features, including federated learning compatibility and on-device processing capabilities, to minimize data sharing requirements in potential future applications, addressing data governance considerations in healthcare artificial intelligence (AI) systems.

The study protocol adheres to the Declaration of Helsinki principles for medical research involving human subjects. All original TCGA data were collected with informed patient consent for research use. No additional ethical approval was required for this computational analysis of publicly available, de-identified data.

### Problem formulation

We formulate multimodal survival prediction as a discrete-time hazard modeling problem that explicitly accommodates missing modalities and cancer-type heterogeneity. For patient $i$ with cancer type $c \in \{1, 2,..., C\}$, let $\mathbf{x}^{(i)} = \{\mathbf{x}^{(i)}_{\mathrm{img}}, \mathbf{x}^{(i)}_{\mathrm{rna}}, \mathbf{x}^{(i)}_{\mathrm{txt}}\}$ represent the multimodal input. $\mathbf{x}^{(i)}_{\mathrm{img}} \in \mathbb{R}^{2048}$ represents histopathological features aggregated from whole slide images (always available). $\mathbf{x}^{(i)}_{\mathrm{rna}} \in \mathbb{R}^{256} \cup \{\emptyset \}$ represents RNA expression features from BulkRNABert embeddings (may be missing). $\mathbf{x}^{(i)}_{\mathrm{txt}} \in \mathbb{R}^{768} \cup \{\emptyset \}$ represents clinical text features from pathology reports (may be missing).

Our objective is to estimate the discrete-time hazard function $h(t|\mathbf{x}^{(i)}, c)$ and derive survival probabilities $S(t|\mathbf{x}^{(i)}, c)$ while explicitly modeling cancer-type heterogeneity and missing data uncertainty.

### Multimodal data preprocessing

#### Whole slide image processing

We adopt a Vision Transformer (ViT) architecture with pretrained weights from Marugoto [36]. Each WSI is divided into nonoverlapping $224 \times 224$ patches, with background patches (entropy $< 5$) discarded. For patient $i$, the raw WSI produces $N_{i}$ patches with features $\mathbf{X}_{\mathrm{raw}}^{(i)} \in \mathbb{R}^{N_{i} \times 2048}$. To obtain a unified representation, we apply K-means clustering with $k = 128$ clusters and use cluster centers as the aggregated representation: 


(1) \begin{align*}& \mathbf{x}^{(i)}_{\mathrm{img}} = \mathrm{KMeans}(\mathbf{X}_{\mathrm{raw}}^{(i)}) \in \mathbb{R}^{2048}\end{align*}

#### RNA expression processing

We use bulk RNA data from TCGA processed through BulkRNABert with pretrained weights [37]. Each RNA sequence generates a 256D embedding capturing molecular expression signatures relevant for survival prediction.

#### Clinical text processing

We collect pathology reports from TCGA and transform them from PDF format to editable text using AWS OCR tools. A text encoder with feature dimension 768 converts raw text into token embeddings that capture clinical characteristics and treatment history.

#### Batch processing details

During training, multimodal data are processed in batches to enable efficient computation. The batch processing dimensions are:

- *Image modality*: Raw patch sequences $\mathbf{X}_{\mathrm{img}}^{\mathrm{batch}} \in \mathbb{R}^{B \times N_{\mathrm{patch}} \times 2048}$, where $N_{\mathrm{patch}} = 128$ represents the number of patches per patient. After mean pooling across patches, we obtain $\mathbf{x}_{\mathrm{img}}^{\mathrm{batch}} \in \mathbb{R}^{B \times 2048}$.
- *Text modality*: Tokenized sequences $\mathbf{X}_{\mathrm{txt}}^{\mathrm{batch}} \in \mathbb{R}^{B \times L_{\mathrm{max}} \times 768}$, where $L_{\mathrm{max}} = 200$ is the maximum sequence length. After mean pooling across tokens, we obtain $\mathbf{x}_{\mathrm{txt}}^{\mathrm{batch}} \in \mathbb{R}^{B \times 768}$.
- *RNA modality*: Gene expression sequences $\mathbf{X}_{\mathrm{rna}}^{\mathrm{batch}} \in \mathbb{R}^{B \times G \times 256}$, where $G = 2048$ represents the number of gene features. After mean pooling across genes, we obtain $\mathbf{x}_{\mathrm{rna}}^{\mathrm{batch}} \in \mathbb{R}^{B \times 256}$.

### Adaptive gated fusion for missing modality scenarios

#### Modality projection and missing data handling

The adaptive gated fusion mechanism operates under a critical constraint: pathological image modality must be available (enforced during dataset preprocessing), while RNA and text modalities may be missing. The system handles this partial modality availability through a two-stage process: modality-specific projection and dynamic weight redistribution. Each available modality is first projected into a unified embedding space of dimension $d = 32$: 


(2) \begin{align*}& \mathbf{Z}_{m}^{\mathrm{batch}} = \mathrm{Dropout}(\mathrm{ReLU}(\mathbf{X}_{m}^{\mathrm{batch}} \mathbf{W}_{m}^{T} + \mathbf{b}_{m})) \in \mathbb{R}^{B \times 32},\end{align*}

where the projection matrices are $\mathbf{W}_{\mathrm{img}} \in \mathbb{R}^{32 \times 2048}$ (always projected as images are mandatory), $\mathbf{W}_{\mathrm{rna}} \in \mathbb{R}^{32 \times 256}$, $\mathbf{W}_{\mathrm{txt}} \in \mathbb{R}^{32 \times 768}$, and $\mathbf{b}_{m} \in \mathbb{R}^{32}$ for each modality $m \in \{\mathrm{img}, \mathrm{rna}, \mathrm{txt}\}$.

For potentially missing RNA and text modalities, the system employs zero-tensor substitution. When modality $m \in \{\mathrm{rna}, \mathrm{txt}\}$ is unavailable for sample $i$, we set 


(3) \begin{align*}& \mathbf{Z}_{m,i}^{\mathrm{batch}} = \mathbf{0} \in \mathbb{R}^{32}\end{align*}

This approach maintains consistent tensor dimensions while allowing the gating mechanism to automatically redistribute attention weights between the mandatory image modality and other available modalities.

#### Dynamic gating with availability masking

The gating mechanism computes attention weights based on the concatenated representations of all modalities. The gate network processes the full multimodal representation: 


(4) \begin{align*}& \mathbf{Z}_{\mathrm{concat}}^{\mathrm{batch}} = \mathrm{Concat}(\mathbf{Z}_{\mathrm{img}}^{\mathrm{batch}}, \mathbf{Z}_{\mathrm{rna}}^{\mathrm{batch}}, \mathbf{Z}_{\mathrm{txt}}^{\mathrm{batch}}) \in \mathbb{R}^{B \times 96}\end{align*}

The raw gate logits are computed as 


(5) \begin{align*}& \mathbf{L}^{\mathrm{batch}} = \mathbf{Z}_{\mathrm{concat}}^{\mathrm{batch}} \mathbf{W}_{\mathrm{gate}}^{T} \in \mathbb{R}^{B \times 3},\end{align*}

where $\mathbf{W}_{\mathrm{gate}} \in \mathbb{R}^{3 \times 96}$.

To handle potentially missing RNA and text modalities, we apply a binary availability mask $\mathbf{M} \in \{0,1\}^{B \times 3}$ where the image position is always 1 while RNA and text positions are set according to actual availability. The key masking strategy is implemented through logit penalties: 


(6) \begin{align*}& \mathbf{L}_{\mathrm{masked}}^{\mathrm{batch}} = \mathbf{L}^{\mathrm{batch}} + (\mathbf{1} - \mathbf{M}) \odot (-1 \times 10^{9})\end{align*}

The use of addition here leverages the mathematical properties of the softmax function: when a logit value becomes extremely small (−1e9), the corresponding probability after softmax normalization approaches zero. This design is more natural than direct zero-assignment because it (i) maintains mathematical consistency with softmax operations; (ii) ensures automatic weight redistribution to available modalities; and (iii) preserves differentiability for end-to-end training

The final attention weights are obtained through softmax normalization: 


(7) \begin{align*}& \mathbf{G}^{\mathrm{batch}} = \mathrm{Softmax}(\mathbf{L}_{\mathrm{masked}}^{\mathrm{batch}}) \in \mathbb{R}^{B \times 3}\end{align*}

This masking strategy ensures that missing RNA or text modalities receive near-zero attention weights, effectively removing them from the fusion process while guaranteeing that the image modality always participates in fusion. The network can dynamically redistribute weights between the image modality and other available modalities.

#### Weighted fusion and output generation

The final fused representation combines the projected modalities using the learned attention weights: 


(8) \begin{align*}& \mathbf{Z}_{\mathrm{fused}}^{\mathrm{batch}} = \sum_{m=1}^{3} \mathbf{G}_{:,m}^{\mathrm{batch}} \odot \mathbf{Z}_{m}^{\mathrm{batch}} \in \mathbb{R}^{B \times 32},\end{align*}

where $\mathbf{G}_{:,m}^{\mathrm{batch}} \in \mathbb{R}^{B \times 1}$ represents the attention weights for modality $m$ across the batch, and $\odot $ denotes element-wise multiplication. This approach enables the model to maintain robust performance even when only images and a subset of other modalities are available, as the attention mechanism automatically adapts to the available information while always ensuring the participation of image information, without requiring explicit imputation strategies.

### Hybrid architecture with cancer-type-specific prediction heads

After gated fusion, features are enhanced through a shared transformer encoder: 


(9) \begin{align*}& \mathbf{H}^{\mathrm{batch}} = \mathrm{GlobalAvgPool}(\mathrm{TransformerEncoder}(\mathbf{Z}_{\mathrm{fused}}^{\mathrm{batch}})) \in \mathbb{R}^{B \times 32}\end{align*}

Cancer-type-specific hazard predictions are generated through dedicated prediction heads. For cancer type $c$ and time bins $T = 4$: 


(10) \begin{align*}& \hat{\mathbf{h}}_{c}^{(i)} = \sigma(\mathbf{W}_{c}^{\mathrm{hazard}} \mathbf{h}^{(i)} + \mathbf{b}_{c}^{\mathrm{hazard}}) \in \mathbb{R}^{4},\end{align*}

where $\mathbf{W}_{c}^{\mathrm{hazard}} \in \mathbb{R}^{4 \times 32}$ and $\mathbf{b}_{c}^{\mathrm{hazard}} \in \mathbb{R}^{4}$ are cancer-type-specific parameters.

For batches containing different cancer types, the model dynamically selects corresponding prediction heads: 


(11) \begin{align*} \hat{\mathbf{h}}_{c_{i}}^{(i)} &= \sigma(\mathbf{W}_{c_{i}}^{\mathrm{hazard}} \mathbf{h}^{(i)} + \mathbf{b}_{c_{i}}^{\mathrm{hazard}}) \in \mathbb{R}^{4}\nonumber\\ \hat{\mathbf{H}}^{\mathrm{batch}} &= [\hat{\mathbf{h}}_{c_{1}}^{(1)}, \hat{\mathbf{h}}_{c_{2}}^{(2)}, \ldots, \hat{\mathbf{h}}_{c_{B}}^{(B)}]^{T} \in \mathbb{R}^{B \times 4},\end{align*}

where $c_{i}$ denotes the cancer type of the $i$th sample, and the model maintains $C = 10$ different sets of prediction head parameters.

The corresponding survival function is 


(12) \begin{align*}& \hat{S}_{c}^{(i)}(t) = \prod_{k=1}^{t} (1 - \hat{h}_{c,k}^{(i)}) \in \mathbb{R}^{4}\end{align*}

### Loss function and evaluation metrics

We optimize using negative log-likelihood loss for discrete-time survival analysis with censored observations: 


(13) \begin{align*}& \mathcal{L} = -\frac{1}{N} \sum_{i=1}^{N} \left[ (1-\delta_{i}) \log S_{\mathrm{prev}}^{(i)} + (1-\delta_{i}) \log h_{\tau_{i}}^{(i)} + \delta_{i} \log S_{\tau_{i}}^{(i)} \right]\end{align*}

For training with batch size $B$, the loss function is computed as 


(14) \begin{align*}& \mathcal{L}_{\mathrm{batch}} = -\frac{1}{B} \sum_{i=1}^{B} \left[ (1-\delta_{i}) \log S_{\mathrm{prev}}^{(i)} + \delta_{i} \log h_{\tau_{i}}^{(i)} + (1-\delta_{i}) \log S_{\tau_{i}}^{(i)} \right],\end{align*}

where each sample’s survival and hazard functions are extracted from the batch prediction results $\hat{\mathbf{H}}^{\mathrm{batch}}$.

Model performance is evaluated using the concordance index (C-index): 


(15) \begin{align*}& \mathrm{C-index} = \frac{\sum_{i,j} \mathbf{1}[t_{i} < t_{j}, \delta_{i} = 1] \cdot \mathbf{1}[\mathrm{risk}_{i}> \mathrm{risk}_{j}]}{\sum_{i,j} \mathbf{1}[t_{i} < t_{j}, \delta_{i} = 1]},\end{align*}

where $\mathbf{1}[\cdot ]$ is the indicator function, providing robust assessment of prognostic accuracy with values ranging from 0.5 (random) to 1.0 (perfect concordance).

## Results

### Experimental framework and evaluation strategy

We evaluated our cancer-type-aware multimodal framework using TCGA data across 10 cancer cohorts with sufficient sample sizes ($>150$ patients): UCEC, LUAD, LGG,BRCA, BLCA, PAAD, COAD, READ, KIRC, and GBM. Three data modalities were included in the model development and analysis: histopathological images, RNA expression profiles, and clinical text records.

**Figure 1:**
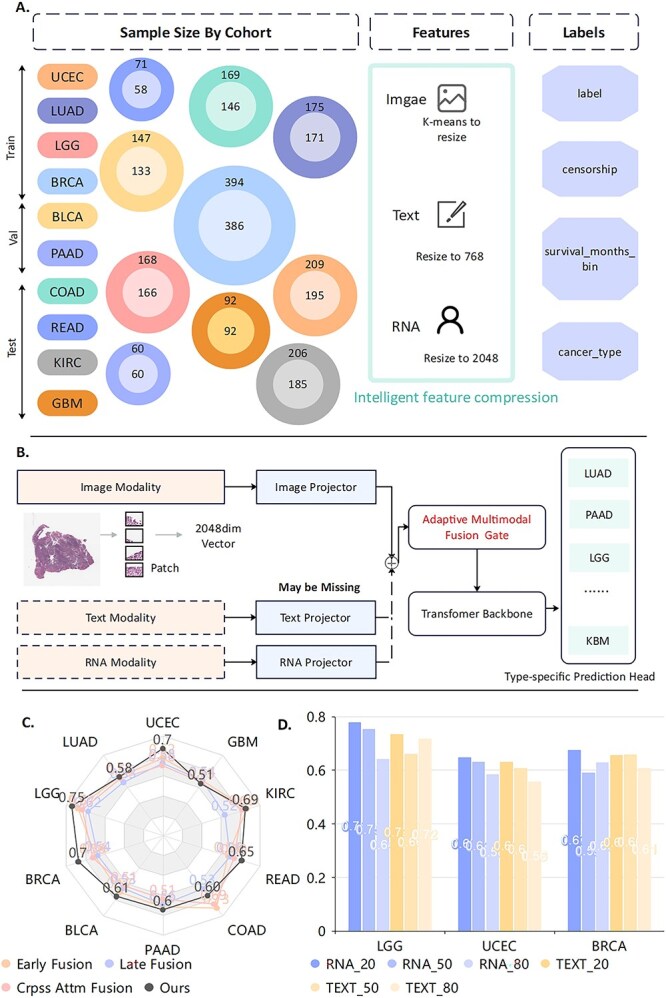
Cancer-type-aware multimodal survival prediction framework and performance showing (A) experimental design across ten cancer types with three modalities, (B) architecture with modality-specific encoders and adaptive gated fusion for missing modalities, (C) C-index comparison with conventional fusion methods, and (D) robustness under varying missing rates for RNA and clinical text modalities.

To assess model robustness under realistic constraints, we designed a comprehensive evaluation protocol encompassing three critical scenarios: (1) cancer type heterogeneity, assessed through independent cohort analysis, (2) incomplete multimodal data, simulated via systematic modality exclusion, and (3) cross-institutional variability, examined using institution-based data partitioning. A data-constrained 4:2:4 train-validation-test split was employed to reflect realistic training data constraints while enabling rigorous evaluation on substantial test cohorts. The sample distribution across splits for each cancer type is shown in Fig. 1A, with our framework architecture illustrated in Fig. 1B.

### From cancer commonality-heterogeneity balance to fusion performance

**Figure 2:**
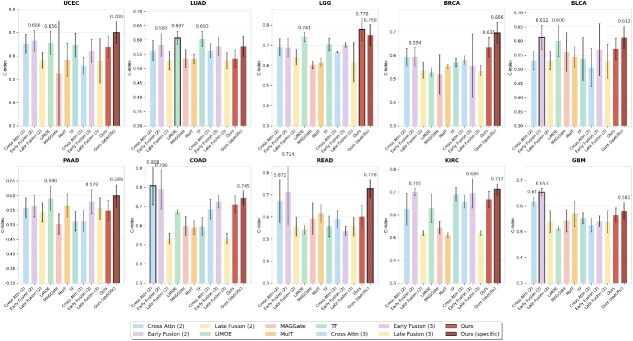
Performance comparison of fusion methods across ten cancer types using the 4:2:4 data split, showing C-index performance with error bars representing standard deviations for twelve fusion approaches including dual-modality methods, full-modality baseline methods, and our proposed framework variants.

We evaluated the performance of our framework in 10 types of cancer (Fig. 2). All baseline methods were trained independently for each cancer type, representing current standard practice. We compared two variants of our approach: **Ours**, which means our framework trained independently per cancer type, and **Ours (specific)**, which employs our hybrid architecture combining shared multimodal encoders with cancer-type-specific prediction heads. Details about the data can be found in the Supplementary Table S1.

The hybrid architecture demonstrated significant improvement over independent training (mean C-index: $0.670 \pm 0.066$ versus $0.625 \pm 0.068$), representing a 7.2% performance gain. Our hybrid cancer-type-specific framework achieved the highest performance in six out of 10 cancer types: UCEC ($0.700 \pm 0.048$), LGG ($0.778 \pm 0.055$), BRCA ($0.696 \pm 0.045$), BLCA ($0.612 \pm 0.040$), PAAD ($0.599 \pm 0.038$), READ ($0.728 \pm 0.041$), and KIRC ($0.712 \pm 0.025$), achieving a mean C-index of $0.670 \pm 0.066$ across all cancer types.

### Robustness under incomplete multimodal data

**Figure 3:**
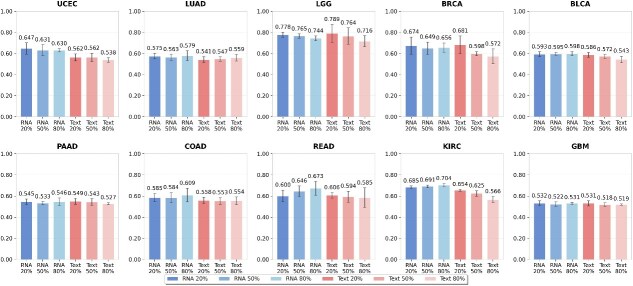
Robustness evaluation under missing modality scenarios across ten cancer types, showing C-index performance with error bars for different missing data rates in RNA and clinical text modalities (20%, 50%, and 80%).

In practice, multimodal data are often incomplete. To evaluate the robustness of our framework under such conditions, we systematically tested its performance in controlled missing-modality scenarios (Fig. 3). During evaluation, auxiliary modalities (RNA expression and clinical text) were randomly excluded for 20%, 50%, or 80% of patients, while histopathological features were consistently retained. Details about the data can be found in the Supplementary Table S2. Our framework demonstrated strong resilience to missing RNA expression data, maintaining mean C-indices of $0.621$, $0.618$, and $0.627$ for 20%, 50%, and 80% missing data, respectively. In missing clinical text scenarios, the framework is slightly more sensitive, with mean C-indices declining from $0.606$ to $0.588$ and $0.568$ as missing data increased from 20% to 80%. Across most cancer types, performance remained robust, with LGG maintaining consistently high C-indices and KIRC showing stable results across all missing-data conditions.

### Stability across institutions

**Figure 4:**
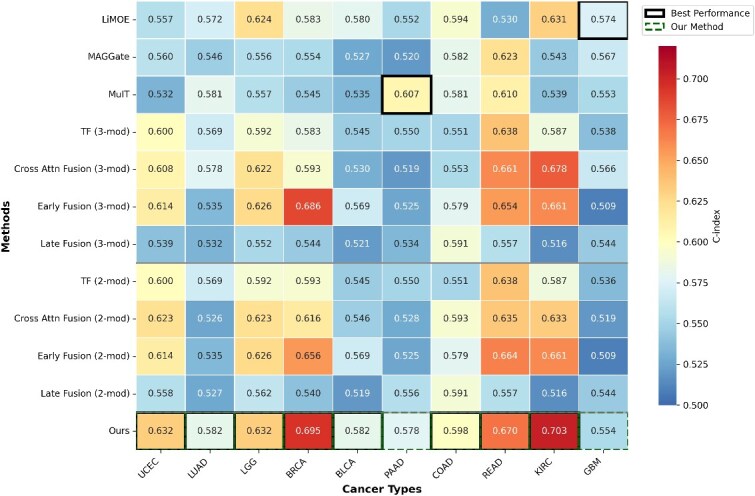
Cross-institutional validation performance across ten cancer types, visualized as a heatmap of C-index values for different fusion methods with the best-performing method highlighted for each cancer type.

Methodological reproducibility requires models to generalize reliably across institutions. To examine this, we conducted cross-institutional validation using TCGA’s multicenter architecture (Fig. 4). Institutional separation was strictly maintained during data partitioning to prevent data leakage and ensure realistic generalization evaluation. Specifically, we assigned entire tissue source sites to train, validate, or test sets, ensuring no patient from the same institution appeared in multiple splits. This institutional-level separation provides more rigorous evaluation than conventional random patient-level splits, as it tests generalization across different imaging scanners, pathology lab protocols, clinical documentation styles, and patient demographics. Details about the institutional partitioning configuration spanning 12–38 contributing centers per cancer type are provided in Supplementary Table S3.

Our multimodal framework achieved state-of-the-art performance among computational baselines across eight cancer types: UCEC ($0.632 \pm 0.017$), LUAD ($0.582 \pm 0.008$), LGG ($0.632 \pm 0.033$), BRCA ($0.695 \pm 0.037$), BLCA ($0.582 \pm 0.007$), COAD ($0.598 \pm 0.064$), READ ($0.670 \pm 0.127$), and KIRC ($0.703 \pm 0.007$). These results are consistent with the performance ranges reported by recent multimodal survival prediction methods using TCGA [10, 11, 39], which typically achieve C-index values similar to us across diverse cancer types when compared against computational baselines.

To ensure robust statistical validation, we repeated all experiments with five independent random seeds (123, 132, 213, 231, 321). Paired t-tests with Bonferroni correction ($\alpha = 0.05/7 = 0.0071$ per cancer type) demonstrated that our method significantly outperforms baseline methods in 42.9% of comparisons (30 out of 70), with particularly strong improvements in BRCA (6/7 baselines), KIRC (5/7 baselines), BLCA (5/7 baselines), and LGG (4/7 baselines). Notably, for BLCA and KIRC, our framework achieved standard deviations of 0.007 across the five runs, demonstrating exceptional consistency.

Regarding variance stability, our method demonstrates numerically lower variance in the majority of comparisons. F-tests comparing variances confirm statistical significance ($P <.05$, $F> 6.39$ for $df_{1}=df_{2}=4$) in select comparisons, particularly in KIRC and LUAD, despite the constrained statistical power of five repetitions. This stability is important for computational reproducibility across sites with varying data collection protocols. Comprehensive statistical testing results, including detailed comparisons for all cancer types and baselines, are provided in Supplementary Tables S5 and S6.

### Data split sensitivity analysis

To assess the framework’s stability under different training-data availability scenarios, we compared data-constrained (4:2:4) and conventional (7:1:2) split strategies across all 10 cancer types (Table 1). Our multimodal framework demonstrated strong stability across both partitioning schemes, with performance variations ranging from 0.006 to 0.153 across cancer types (mean absolute difference: 0.048 $\pm $ 0.041). Notably, nine out of 10 cancer types showed performance changes below 0.070, indicating minimal sensitivity to training set size. The largest variation was observed in COAD (0.153 increase with 4:2:4 split), likely reflecting the benefit of larger test sets for more robust evaluation in cancers with high biological heterogeneity.

In contrast, baseline methods exhibited substantially greater instability when evaluated under reduced training data conditions. For instance, MAGGate showed a 0.120 decrease in GBM performance when switching from 7:1:2 to 4:2:4 splits, while Early Fusion methods demonstrated inconsistent behavior across cancer types with changes ranging from −0.094 to +0.138. This instability suggests that conventional fusion approaches are highly dependent on abundant training data and may not generalize reliably when applied in data-constrained settings.

## Discussion

This work presents a computational methods contribution that advances multimodal fusion techniques for survival prediction under realistic data constraints. We explicitly clarify that this study does not claim readiness for clinical deployment, propose replacement of standard-of-care prognostic tools (e.g. TNM staging, clinical nomograms), or demonstrate incremental clinical utility. Rather, we address three fundamental technical prerequisites—(i) handling missing modalities without imputation, (ii) modeling cancer-type-specific heterogeneity, and (iii) ensuring cross-institutional stability—that must be solved before multimodal AI models can be considered for prospective clinical validation.

Our evaluation framework compares against 12 state-of-the-art computational methods across 10 cancer types with institutional-level cross-validation, representing the appropriate benchmark for computational methods papers in medical AI and bioinformatics. This evaluation approach is consistent with highly cited methodological work in this domain [10, 11, 39], all of which benchmark against computational baselines rather than clinical staging systems. Direct comparison with clinical staging would require prospective clinical trials with treatment decision pathways—an entirely different research scope requiring multi-year clinical collaborations, institutional review board approvals, and patient recruitment protocols that extend far beyond computational methods development.

This study presents a cancer-type-aware multimodal framework designed to overcome three major computational obstacles: incomplete multimodal data, tumor-specific biological heterogeneity, and cross-institutional variability. Through deliberate architectural design and comprehensive evaluation across 10 cancer types, we demonstrate how these challenges can be systematically addressed at the computational level to enable more robust and reproducible prognostic modeling.

A primary contribution of this work is the development of an adaptive gated fusion mechanism that robustly handles incomplete multimodal inputs without requiring imputation or distributional assumptions. By dynamically reweighting modalities based on available prognostic signals, the model naturally adapts to scenarios where certain modalities—particularly RNA expression—are systematically missing or inconsistently available. Controlled missing-modality experiments (Fig. 3) demonstrate that the framework maintains performance within established ranges for computational prognostic models [40, 41] with minimal degradation (typically $<7\%$) when RNA expression is absent, the most common constraint in computational oncology datasets. Unlike prior methods that rely on generative imputation or disentanglement [20], our approach eliminates strong distributional assumptions and computational overhead during inference, providing greater flexibility for arbitrary missing patterns.

Our hybrid architecture, integrating shared multimodal encoders with cancer-type-specific prediction heads, provides a principled solution to the trade-off between generalization and specialization. The shared encoder captures global prognostic patterns across malignancies, while cancer-specific heads model the unique pathobiology and survival dynamics of each cancer type. This design achieves state-of-the-art performance among computational baselines in six of 10 cancer types, with notable gains in cancers with well-characterized prognostic patterns—such as LGG (C-index: 0.778) and endometrial carcinoma (UCEC, C-index: 0.700). These results support our hypothesis that cancer-specific modeling captures prognostic signals overlooked by uniform architectures.

Our overall mean C-index of 0.670 $\pm $ 0.066 is consistent with state-of-the-art computational methods in multimodal survival prediction. For comparison, Chen *et al*. [39] reported C-index values of $\sim $0.64 across TCGA cancers, Jaume *et al*. [10] achieved C-index 0.60–0.68 across five cancer types, and Song *et al*. [11] demonstrated similar performance ranges. All of these benchmark works evaluated their methods exclusively against computational baselines using TCGA data, establishing this as the standard evaluation paradigm for methodological contributions in this domain. Establishing clinical utility beyond these computational benchmarks requires prospective validation studies demonstrating impact on treatment decisions and patient outcomes compared with standard-of-care prognostic assessments—a critical next step that represents a fundamentally different research scope requiring clinical trial infrastructure.

Cross-institutional validation further demonstrates the framework’s computational stability in heterogeneous data environments. The consistently low performance variance (standard deviations < 0.040 across 12–38 institutions) indicates strong resilience to differences in imaging protocols, reporting styles, and institutional patient populations. This stability is essential for methodological reproducibility across sites, addressing a key limitation identified in prior work [34]. Two design decisions were particularly impactful: (i) anchoring the model on histopathology—the universally available modality across oncology workflows—ensures baseline functionality even when auxiliary modalities are unavailable, and (ii) incorporating structured modality dropout during training exposes the model to diverse missing-data patterns and improves generalization. Additionally, our cancer-type-specific modeling approach reduces overfitting that can occur when forcing heterogeneous cancer types into a single unified architecture.

Analysis of data split strategies (Table 1) reveals strong computational stability under varying training data availability. The data-constrained 4:2:4 split demonstrates performance variations of 0.006–0.153 across cancer types (mean: $0.048 \pm 0.041$), with nine of 10 cancers showing changes below 0.070. In contrast, baseline methods optimized for abundant training data frequently fail under reduced training scenarios, exhibiting substantially greater instability. This robustness addresses reproducibility concerns for application across institutions with different data collection capabilities.

**Table 1:** Model stability analysis across different data splits (7:1:2 vs. 4:2:4) for all cancer types, reporting C-index performance and absolute performance changes for different multimodal fusion methods.

**Method**	**Split**	**UCEC**	**LUAD**	**LGG**	**BRCA**	**BLCA**	**PAAD**	**COAD**	**READ**	**KIRC**	**GBM**
**Three modality methods (text, RNA, image)**											
LiMOE	7:1:2	0.619$\pm $0.096	0.585$\pm $0.015	0.685$\pm $0.018	0.673$\pm $0.007	0.594$\pm $0.010	0.568$\pm $0.030	0.615$\pm $0.034	0.525$\pm $0.065	0.667$\pm $0.012	0.543$\pm $0.033
	4:2:4	0.656$\pm $0.050	**0.617** $\pm $ **0.024**	0.741$\pm $0.023	0.529$\pm $0.018	0.600$\pm $0.055	0.590$\pm $0.042	0.672$\pm $0.010	0.542$\pm $0.019	0.629$\pm $0.062	0.513$\pm $0.009
		($\uparrow $0.037)	($\uparrow $0.032)	($\uparrow $0.056)	($\downarrow $0.144)	($\uparrow $0.006)	($\uparrow $0.022)	($\uparrow $0.057)	($\uparrow $0.017)	($\downarrow $0.038)	($\downarrow $0.030)
MAGGate	7:1:2	0.562$\pm $0.051	0.542$\pm $0.030	0.599$\pm $0.059	0.535$\pm $0.020	0.557$\pm $0.032	0.570$\pm $0.063	0.570$\pm $0.041	0.592$\pm $0.067	0.526$\pm $0.015	**0.663** $\pm $ **0.088**
	4:2:4	0.525$\pm $0.023	0.535$\pm $0.031	0.603$\pm $0.019	0.519$\pm $0.085	0.561$\pm $0.068	0.502$\pm $0.035	0.599$\pm $0.049	0.593$\pm $0.071	0.544$\pm $0.028	0.543$\pm $0.043
		($\downarrow $0.037)	($\downarrow $0.007)	($\uparrow $0.004)	($\downarrow $0.016)	($\uparrow $0.004)	($\downarrow $0.068)	($\uparrow $0.029)	($\uparrow $0.001)	($\uparrow $0.018)	($\downarrow $0.120)
MuIT	7:1:2	0.553$\pm $0.019	0.572$\pm $0.014	0.650$\pm $0.013	0.568$\pm $0.027	0.537$\pm $0.029	0.605$\pm $0.046	0.544$\pm $0.017	0.616$\pm $0.040	0.554$\pm $0.044	0.614$\pm $0.078
	4:2:4	0.584$\pm $0.071	0.534$\pm $0.017	0.614$\pm $0.019	0.553$\pm $0.008	0.544$\pm $0.037	0.566$\pm $0.040	0.590$\pm $0.035	0.616$\pm $0.040	0.511$\pm $0.010	0.570$\pm $0.049
		($\uparrow $0.031)	($\downarrow $0.038)	($\downarrow $0.036)	($\downarrow $0.015)	($\uparrow $0.007)	($\downarrow $0.039)	($\uparrow $0.046)	($\uparrow $0.000)	($\downarrow $0.043)	($\downarrow $0.044)
TF	7:1:2	0.636$\pm $0.044	**0.600** $\pm $ **0.042**	0.710$\pm $0.064	0.591$\pm $0.036	0.581$\pm $0.030	0.562$\pm $0.038	**0.654** $\pm $ **0.052**	0.544$\pm $0.032	**0.743** $\pm $ **0.029**	0.575$\pm $0.030
	4:2:4	0.647$\pm $0.051	0.603$\pm $0.026	0.704$\pm $0.031	0.570$\pm $0.018	0.537$\pm $0.055	0.512$\pm $0.033	0.595$\pm $0.049	0.558$\pm $0.048	0.690$\pm $0.030	0.552$\pm $0.021
		($\uparrow $0.011)	($\uparrow $0.003)	($\downarrow $0.006)	($\downarrow $0.021)	($\downarrow $0.044)	($\downarrow $0.050)	($\downarrow $0.059)	($\uparrow $0.014)	($\downarrow $0.053)	($\downarrow $0.023)
Cross Attn	7:1:2	0.623$\pm $0.032	0.526$\pm $0.015	0.623$\pm $0.036	0.616$\pm $0.063	0.546$\pm $0.025	0.528$\pm $0.022	0.613$\pm $0.135	0.635$\pm $0.152	0.633$\pm $0.075	0.519$\pm $0.015
Fusion	4:2:4	0.558$\pm $0.035	0.563$\pm $0.025	0.667$\pm $0.004	0.580$\pm $0.018	0.506$\pm $0.027	0.513$\pm $0.035	0.686$\pm $0.051	0.590$\pm $0.038	0.658$\pm $0.027	0.526$\pm $0.027
		($\downarrow $0.065)	($\uparrow $0.037)	($\uparrow $0.044)	($\downarrow $0.036)	($\downarrow $0.040)	($\downarrow $0.015)	($\uparrow $0.073)	($\downarrow $0.045)	($\uparrow $0.025)	($\uparrow $0.007)
Early Fusion	7:1:2	0.625$\pm $0.026	0.522$\pm $0.027	0.753$\pm $0.015	0.641$\pm $0.016	0.560$\pm $0.029	0.562$\pm $0.028	0.619$\pm $0.035	0.607$\pm $0.040	0.721$\pm $0.015	0.635$\pm $0.068
	4:2:4	0.622$\pm $0.050	0.578$\pm $0.031	0.701$\pm $0.011	0.556$\pm $0.034	0.569$\pm $0.022	0.579$\pm $0.040	0.726$\pm $0.033	0.537$\pm $0.020	0.695$\pm $0.064	0.541$\pm $0.021
		($\downarrow $0.003)	($\uparrow $0.056)	($\downarrow $0.052)	($\downarrow $0.085)	($\uparrow $0.009)	($\uparrow $0.017)	($\uparrow $0.107)	($\downarrow $0.070)	($\downarrow $0.026)	($\downarrow $0.094)
Late Fusion	7:1:2	0.560$\pm $0.023	0.538$\pm $0.019	0.630$\pm $0.049	0.553$\pm $0.031	0.540$\pm $0.025	0.545$\pm $0.040	0.543$\pm $0.050	0.596$\pm $0.047	0.551$\pm $0.044	0.550$\pm $0.045
	4:2:4	0.579$\pm $0.025	0.528$\pm $0.028	0.617$\pm $0.019	0.535$\pm $0.022	0.529$\pm $0.030	0.556$\pm $0.038	0.533$\pm $0.027	0.556$\pm $0.043	0.520$\pm $0.010	0.539$\pm $0.043
		($\uparrow $0.019)	($\downarrow $0.010)	($\downarrow $0.013)	($\downarrow $0.018)	($\downarrow $0.011)	($\uparrow $0.011)	($\downarrow $0.010)	($\downarrow $0.040)	($\downarrow $0.031)	($\downarrow $0.011)
**Two modality methods (text, image)**											
Cross Attn	7:1:2	0.630$\pm $0.008	0.519$\pm $0.012	0.747$\pm $0.018	0.659$\pm $0.057	0.549$\pm $0.018	**0.620** $\pm $ **0.028**	0.614$\pm $0.039	0.656$\pm $0.024	0.707$\pm $0.018	0.599$\pm $0.022
Fusion	4:2:4	0.651$\pm $0.040	0.563$\pm $0.035	0.691$\pm $0.042	0.593$\pm $0.038	0.531$\pm $0.032	0.558$\pm $0.035	**0.808** $\pm $ **0.100**	0.671$\pm $0.097	0.625$\pm $0.070	0.617$\pm $0.018
		($\uparrow $0.021)	($\uparrow $0.044)	($\downarrow $0.056)	($\downarrow $0.066)	($\downarrow $0.018)	($\downarrow $0.062)	($\uparrow $0.194)	($\uparrow $0.015)	($\downarrow $0.082)	($\uparrow $0.018)
Early Fusion	7:1:2	0.624$\pm $0.020	0.528$\pm $0.021	0.751$\pm $0.009	0.635$\pm $0.015	0.558$\pm $0.022	0.590$\pm $0.036	0.652$\pm $0.028	0.585$\pm $0.026	0.714$\pm $0.009	0.591$\pm $0.024
	4:2:4	0.666$\pm $0.045	0.583$\pm $0.040	0.688$\pm $0.044	0.594$\pm $0.039	**0.612** $\pm $ **0.042**	0.565$\pm $0.038	0.790$\pm $0.102	**0.714** $\pm $ **0.149**	0.701$\pm $0.016	**0.653** $\pm $ **0.015**
		($\uparrow $0.042)	($\uparrow $0.055)	($\downarrow $0.063)	($\downarrow $0.041)	($\uparrow $0.054)	($\downarrow $0.025)	($\uparrow $0.138)	($\uparrow $0.129)	($\downarrow $0.013)	($\uparrow $0.062)
Late Fusion	7:1:2	0.562$\pm $0.020	0.547$\pm $0.037	0.640$\pm $0.024	0.559$\pm $0.020	0.537$\pm $0.029	0.560$\pm $0.036	0.556$\pm $0.025	0.589$\pm $0.065	0.526$\pm $0.012	0.576$\pm $0.033
	4:2:4	0.582$\pm $0.035	0.528$\pm $0.032	0.641$\pm $0.038	0.537$\pm $0.034	0.531$\pm $0.032	0.543$\pm $0.033	0.533$\pm $0.027	0.556$\pm $0.043	0.520$\pm $0.010	0.539$\pm $0.043
		($\uparrow $0.020)	($\downarrow $0.019)	($\uparrow $0.001)	($\downarrow $0.022)	($\downarrow $0.006)	($\downarrow $0.017)	($\downarrow $0.023)	($\downarrow $0.033)	($\downarrow $0.006)	($\downarrow $0.037)
**Ours**	7:1:2	**0.666** $\pm $ **0.040**	0.545$\pm $0.009	**0.769** $\pm $ **0.011**	**0.719** $\pm $ **0.024**	**0.606** $\pm $ **0.010**	0.548$\pm $0.020	0.592$\pm $0.024	**0.659** $\pm $ **0.061**	0.700$\pm $0.013	0.574$\pm $0.011
	4:2:4	**0.700** $\pm $ **0.028**	0.578$\pm $0.025	**0.750** $\pm $ **0.012**	**0.696** $\pm $ **0.025**	0.612$\pm $0.020	**0.599** $\pm $ **0.038**	0.745$\pm $0.038	**0.728** $\pm $ **0.041**	**0.712** $\pm $ **0.025**	0.581$\pm $0.032
		($\uparrow $0.034)	($\uparrow $0.033)	($\downarrow $0.019)	($\downarrow $0.023)	($\uparrow $0.006)	($\uparrow $0.051)	($\uparrow $0.153)	($\uparrow $0.069)	($\uparrow $0.012)	($\uparrow $0.007)

The differential impact of missing RNA expression versus missing clinical text (Fig. 3) demonstrates computational robustness patterns that align with empirical observations in cancer prognosis research. RNA expression provides substantial prognostic value even with 80% missingness, reflecting its established importance in computational prognostic models. Cancer types with documented molecular stratification (LGG, KIRC) maintain high performance even without RNA, indicating that histopathological features contain sufficient prognostic signals in these computational models. In contrast, cancers with more heterogeneous histological patterns (GBM, PAAD) show greater performance benefit from multimodal integration. While these computational patterns are consistent with empirical observations reported in cancer prognosis research, formal validation through attention analysis on pathologist-annotated regions would constitute separate biological discovery work requiring pathologist collaboration.

Despite these computational advances, several limitations warrant consideration. First, all analyses were performed on TCGA, a curated research dataset widely used as a benchmark for computational pathology methods [35, 42]. Our institutional-level cross-validation across 12–38 TCGA contributing centers provides evidence of generalizability across heterogeneous data collection protocol, offering a more complementary evaluation perspective to the random patient-level splits used in most prior work [39]. However, external validation on independent hospital datasets remains an important next step. Such validation will likely require multi-institutional collaborations, data sharing agreements, and standardized clinical baseline comparisons, which together form necessary—though not sufficient—technical foundations for prospective clinical validation and eventual clinical translation. Second, although our model is robust to missing modalities during inference, it still requires complete multimodal data during training. Meta-learning or self-supervised approaches enabling flexible training under incomplete data regimes could further enhance applicability, particularly for rare cancer types with limited complete-case samples. Third, interpretability remains limited. While cancer-specific prediction heads provide some insight into tumor-specific prognostic patterns, more systematic attribution approaches—such as attention visualization, feature importance analysis, and integration with known biological markers—could improve transparency Fourth, this study provides a computational foundation addressing key technical prerequisites—including missing-data robustness, cancer heterogeneity modeling, and cross-institutional stability—necessary before rigorous prospective clinical evaluation. Demonstrating incremental clinical benefit over standard prognostic tools will require dedicated clinical studies beyond the scope of this methodological work. Finally, the observation that certain cancer types may benefit from alternative fusion strategies in some scenarios (Fig. 2) suggests opportunities for adaptive ensemble approaches that dynamically select optimal fusion mechanisms, albeit with increased complexity.

In summary, this work provides methodological advances for multimodal survival prediction under realistic constraints. By jointly addressing missing-data robustness, cancer-type heterogeneity, and institutional variability, our framework moves beyond idealized multimodal assumptions toward computational methods that address practical implementation challenges. The robust performance across diverse cancers and data scenarios demonstrates meaningful progress in computational methodology, establishing technical foundations necessary—though not sufficient—for future clinical translation. Future work should prioritize prospective clinical validation, external cohort testing, enhanced interpretability methods, and integration with established clinical decision support systems to fully realize the translational potential of multimodal AI in precision oncology.

**Key Points**

- We developed a cancer-type-aware multimodal framework that maintains robust cancer survival prediction (C-index 0.568–0.627) even with 80% missing auxiliary modalities.
- Our hybrid architecture combining shared encoders with cancer-specific prediction heads achieves 7.2% performance improvement over independent training (mean C-index: 0.670$\pm $0.066 versus 0.625$\pm $0.068), with state-of-the-art results in six out of 10 cancer types.
- Cross-institutional validation across 12–38 centers per cancer type demonstrated strong cross-site stability (standard deviations <0.040 in eight of 10 cancer types), achieved without institution-specific fine-tuning.
- By establishing histopathology as the mandatory anchor modality while adaptively incorporating RNA expression and clinical text through gated fusion, our framework provides consistent base functionality across diverse institutional contexts.
- This work addresses three fundamental technical challenges—incomplete data, cancer heterogeneity, and cross-institutional variability—that represent essential computational prerequisites for future clinical translation of multimodal survival prediction models.

## Supplementary Material

BIB_CancerMoE_supp_v2_bbag124

## Data Availability

The Cancer Genome Atlas (TCGA) data used in this study are publicly available through the Genomic Data Commons (GDC) Data Portal (https://portal.gdc.cancer.gov/) and the National Cancer Institute’s GDC Legacy Archive. Specifically, we utilized whole-slide histopathology images, RNA-seq expression data, and clinical text records for 10 cancer types: UCEC, LUAD, LGG, BRCA, BLCA, PAAD, COAD, READ, KIRC, and GBM. Access to TCGA data requires registration and compliance with the GDC Data Access Policies. The processed multimodal features and survival annotations used for model training and evaluation are available upon reasonable request to the corresponding author, subject to TCGA data use agreements and institutional review board approval. Due to patient privacy considerations and TCGA consortium guidelines, individual-level data cannot be directly shared. Still, researchers can access the same underlying datasets through the official GDC portal using the case identifiers provided in our supplementary materials. The source code for our cancer-type-aware multimodal survival prediction framework is publicly available on GitHub at https://github.com/zongzi13545329/NPJ.git. The repository includes the complete PyTorch implementation along with detailed documentation, configuration files, and comprehensive scripts to reproduce experimental results presented in this study. The codebase supports all experimental settings, including complete and missing modality scenarios, cross-institutional validation, and cancer-type-specific training. Additionally, we provide utilities for statistical analysis, performance evaluation, and full reproducibility through the use of fixed random seeds and deterministic training procedures. The repository also includes the BLCA dataset as an example, with additional cancer datasets to be released subsequently.
