# Supplementary material for: A cancer-type-aware framework for robust multimodal survival prediction under missing modalities
Source: Brief Bioinform. 2026 Mar 23;27(2):bbag124. doi: 10.1093/bib/bbag124 (PMC13006980; doi:10.1093/bib/bbag124)
Supplement: BIB_CancerMoE_supp_v2_bbag124 [file bib_cancermoe_supp_v2_bbag124.pdf]

BRIEFINGS IN BIOINFORMATION, 2022, pp. 1–5

doi: DOI HERE

Advance Access Publication Date: Day Month Year

Paper

PAPER

Supplementary Table Descriptions

A Cancer-Type-Aware Framework for Robust Multimodal Survival Prediction under Missing Modalities

Manuscript ID: BIB-25-2397

Abstract

Supplementary Table Descriptions

Supplementary Table S1 presents a comprehensive comparison of multimodal fusion methods across ten cancer types using a systematic 4:2:4 data split strategy. The table demonstrates the superior performance of our cancer-type-aware framework, achieving state-of-the-art results in seven out of ten cancer types. Our hybrid architecture with cancer-type-specific prediction heads (Ours specific) outperforms both dual-modality and full-modality baseline approaches, validating the effectiveness of adaptive gated fusion and tumor-specific modeling.

Supplementary Table S2 evaluates framework robustness under controlled missing modality scenarios, critical for clinical deployment where complete multimodal data may be unavailable. The systematic evaluation removes RNA expression or clinical text at 20%, 50%, and 80% rates while maintaining histopathological images as the primary modality. Results demonstrate remarkable resilience to missing RNA data with minimal performance degradation, while showing greater sensitivity to missing clinical text. These findings validate practical applicability in healthcare settings with data availability constraints.

Supplementary Table S3 Institutional-level data partitioning configuration across ten TCGA cancer types. Data partitioning was performed strictly according to TCGA Tissue Source Site (TSS) codes, which uniquely identify the contributing institution embedded as the second segment of the TCGA patient barcode [1, 2]. All patients from the same TSS were assigned exclusively to the same split (training, validation, or test) to prevent data leakage and ensure complete independence between splits. This institutional-level separation, maintained across 12–38 contributing centers per cancer type, provides a more stringent evaluation than conventional random patient-level splits, as it tests model generalizability across different imaging scanners, pathology lab protocols, clinical documentation styles, and patient demographics. The table reports: (1) cancer type, (2) data split (train/valid/test), (3) TSS codes assigned to each split, (4) number of institutions per split, and (5) number of patients per split. The 4:2:4 split ratio was designed to reflect realistic scenarios where models must be developed with limited training data while maintaining robust evaluation on substantial test cohorts.

Supplementary Table S4 presents the cross-institutional validation performance across TCGA contributing centers. The table reports concordance index (C-index) with standard deviations for different fusion methods across ten cancer types, following the institutional-level data partitioning described in Supplementary Table S3. Standard deviations were calculated across 5 independent runs with

© The Author 2022. Published by Oxford University Press. All rights reserved. For permissions, please e-mail: journals.permissions@oup.com

different random seeds, each using institutional-level cross-validation as specified in the Methods section. Our framework achieves state-of-the-art performance among computational baselines in eight cancer types (UCEC, LUAD, LGG, BRCA, BLCA, COAD, READ, KIRC) with consistently low standard deviations (typically <0.040), demonstrating computational robustness across diverse institutional settings with varying data collection protocols.

Supplementary Table S5 reports the statistical significance of performance differences and variance stability between our method and seven baseline approaches across ten cancer types. Performance differences were assessed using two-tailed paired t-tests with Bonferroni correction applied within each cancer type to account for 7 pairwise comparisons ($\alpha = 0.05/7 = 0.0071$) across 5 independent runs. Each cell presents the mean C-index difference (our method minus baseline) and the percentage variance reduction. The results demonstrate that our method achieves statistically significant improvements (**bold**, $p < \alpha$) in the majority of comparisons. The most pronounced gains are observed in BRCA (mean difference up to +0.151), KIRC (up to +0.187), and BLCA (up to +0.061). Furthermore, our approach shows exceptional stability in KIRC and LUAD, with variance reductions frequently exceeding 80% and reaching up to 97% compared to most baselines. In certain high-heterogeneity cohorts, such as PAAD and GBM, while our method remains competitive, specific baselines (e.g., MuIT in PAAD and LiMOE in GBM) occasionally yield higher mean performance, as indicated by the † marker. Significant variance expansion (noted as $> 10\times$) in READ and COAD cohorts likely reflects the inherent challenges of cross-institutional validation in small-sample settings. Overall, the statistical evidence confirms that our proposed architecture provides a robust and superior fusion strategy for pan-cancer survival analysis.

Statistical Stability Analysis (Supplementary Table S6): Supplementary Table S6 presents the F-test statistics evaluating the variance stability of our proposed method against seven baseline approaches. The F-statistic, defined as $F = \text{Var(Baseline)}/\text{Var(Ours)}$, quantifies the relative consistency of model performance across 5 independent random seeds ($df_1 = 4, df_2 = 4$). Significance was assessed at $\alpha = 0.05$ ($F > 6.39$) and $\alpha = 0.01$ ($F > 15.98$). The results indicate that our method exhibits significantly higher stability ($F > 1$) in the majority of large-scale cohorts. Specifically, in **KIRC**, our method achieves F-statistics of 47.02 and 39.51 (both $p < 0.01$) against LiMOE and MAGGate, respectively, representing a substantial reduction in performance fluctuation. Similar robust stability is observed in **LUAD**, where all baselines exhibit $3\times$ to $29\times$ higher variance compared to our approach, with four comparisons reaching statistical significance. In the **BLCA** cohort, our method significantly outperforms the TF ($F = 8.16$, $p < 0.05$) and Late Fusion ($F = 7.37$, $p < 0.05$) baselines in terms of predictive reliability. Conversely, in cohorts characterized by limited sample sizes or high clinical heterogeneity (e.g., READ, GBM, and PAAD), F-statistics below 1.0 were observed. This suggests that while our method remains competitive in mean performance, it may exhibit higher sensitivity to specific institutional data distributions during cross-validation in data-sparse regimes. These findings underscore the critical importance of balancing both raw performance and variance stability in pan-cancer multi-modal fusion.

Table 1. Supplementary Table S1. Performance comparison across ten cancer types using 4:2:4 data split. Methods are grouped by modality usage, with dual-modality and full-modality fusion approaches. Best performance per cancer type is highlighted in bold. Results show mean C-index values with standard deviations.

	Cancer Types and Performance Metrics									
Method	UCEC	LUAD	LGG	BRCA	BLCA	PAAD	COAD	READ	KIRC	GBM
**Dual-modality models**										
Cross Attn Fusion	0.651±0.040	0.563±0.035	0.691±0.042	0.593±0.038	0.531±0.032	0.558±0.035	**0.808±0.100**	0.671±0.097	0.625±0.070	0.617±0.018
Early Fusion	0.666±0.045	0.583±0.040	0.688±0.044	0.594±0.039	0.612±0.042	0.565±0.038	0.790±0.102	0.714±0.149	0.701±0.016	**0.653±0.015**
Late Fusion	0.582±0.035	0.528±0.032	0.641±0.038	0.537±0.034	0.531±0.032	0.543±0.033	0.533±0.027	0.556±0.043	0.520±0.010	0.539±0.043
**Full-modality fusion models**										
LiMOE	0.656±0.050	**0.607±0.024**	0.741±0.023	0.529±0.018	0.600±0.055	0.590±0.042	0.672±0.010	0.542±0.019	0.629±0.062	0.513±0.009
MAGGate	0.525±0.223	0.535±0.031	0.603±0.019	0.519±0.085	0.561±0.068	0.502±0.035	0.599±0.049	0.593±0.071	0.544±0.028	0.543±0.043
MuIT	0.584±0.071	0.534±0.017	0.614±0.019	0.553±0.008	0.544±0.037	0.566±0.040	0.590±0.035	0.616±0.040	0.511±0.010	0.570±0.049
TF	0.647±0.051	0.603±0.026	0.704±0.031	0.570±0.018	0.537±0.075	0.512±0.033	0.595±0.049	0.558±0.048	0.690±0.030	0.552±0.021
Cross Attn Fusion	0.558±0.035	0.563±0.025	0.667±0.004	0.580±0.018	0.506±0.067	0.513±0.035	0.686±0.051	0.590±0.038	0.658±0.027	0.526±0.027
Early Fusion	0.622±0.050	0.578±0.031	0.701±0.011	0.556±0.134	0.569±0.092	0.579±0.040	0.726±0.033	0.537±0.020	0.695±0.064	0.541±0.021
Late Fusion	0.579±0.095	0.528±0.028	0.617±0.099	0.535±0.022	0.529±0.060	0.556±0.038	0.533±0.027	0.556±0.043	0.520±0.010	0.539±0.043
**Ours**	0.638±0.045	0.535±0.032	**0.778±0.055**	0.635±0.042	0.572±0.038	0.548±0.035	0.710±0.045	0.601±0.052	0.668±0.035	0.565±0.028
**Ours (specific)**	**0.700±0.048**	0.578±0.035	0.750±0.052	**0.696±0.045**	**0.612±0.040**	**0.599±0.038**	0.745±0.038	**0.728±0.041**	**0.712±0.025**	0.581±0.032

Table 2. Supplementary Table S2. Robustness evaluation under missing modality scenarios. Performance metrics represent mean C-index across all cancer types under different missing data rates for RNA and text modalities. Results demonstrate the clinical applicability of our approach when complete multimodal data is unavailable.

Scenario	UCEC	LUAD	LGG	BRCA	BLCA	PAAD	COAD	READ	KIRC	GBM
RNA 20%	0.647±0.053	0.575±0.026	0.778±0.024	0.674±0.081	0.593±0.023	0.545±0.027	0.585±0.039	0.600±0.056	0.685±0.011	0.532±0.022
RNA 50%	0.631±0.052	0.563±0.027	0.765±0.020	0.649±0.059	0.595±0.014	0.533±0.014	0.584±0.049	0.646±0.050	0.691±0.013	0.522±0.022
RNA 80%	0.630±0.016	0.579±0.046	0.744±0.022	0.656±0.043	0.598±0.020	0.546±0.036	0.609±0.065	0.673±0.066	0.704±0.014	0.531±0.008
Text 20%	0.562±0.033	0.541±0.029	0.789±0.086	0.681±0.085	0.586±0.024	0.549±0.028	0.558±0.030	0.606±0.026	0.654±0.009	0.531±0.025
Text 50%	0.562±0.041	0.547±0.021	0.764±0.078	0.598±0.020	0.572±0.018	0.543±0.031	0.553±0.032	0.594±0.052	0.625±0.026	0.518±0.017
Text 80%	0.538±0.022	0.559±0.031	0.716±0.052	0.572±0.070	0.543±0.032	0.527±0.010	0.554±0.037	0.585±0.093	0.566±0.029	0.519±0.006

References

1. Genomic Data Commons. TCGA Barcode. https://docs.gdc.cancer.gov/Encyclopedia/pages/TCGA_Barcode/, 2024. Accessed: 2025-01-20.
2. Jianfang Liu, Tara Lichtenberg, Katherine A Hoadley, Laila M Poisson, Alexander J Lazar, Andrew D Cherniack, Albert J Kovatich, Christopher C Benz, Douglas A Levine, Adrian V Lee, et al. An integrated tcga pan-cancer clinical data resource to drive high-quality survival outcome analytics. *Cell*, 173(2):400–416, 2018.

Table 3. Supplementary Table S3. Institutional-level data partitioning configuration across ten TCGA cancer types. This institutional-level separation, maintained across 12–38 contributing centers per cancer type, provides a more stringent evaluation than conventional random patient-level splits, as it tests model generalizability across different imaging scanners, pathology lab protocols, clinical documentation styles, and patient demographics.

Cancer	Split	TSS Codes	# Inst.	# Patients
UCEC	Train	BG,DI,BK,K6,2E,D1,EO,DF,QF,5B,5S,EC,KJ,QS,PG,SJ,JU,E6,H5	19	195 (43%)
	Valid	EY,AJ,FI	3	90 (20%)
	Test	AP,A5,B5,BS,AX	5	209 (46%)
LUAD	Train	NJ,78,J2,83,55,62,O1,L4,69,80,4B,73,99,S2	14	171 (40%)
	Valid	05,49,67,35,38,L9,91	7	86 (20%)
	Test	44,95,MN,97,86,93,53,64,75,MP,50	11	175 (40%)
LGG	Train	HT,S9,CS,TQ,KT,HW,W9,EZ,DH	9	166 (40%)
	Valid	QH,DB,P5,RY,WH,VV,FN,IK	8	81 (20%)
	Test	DU,E1,TM,WY,R8,VM,FG	7	168 (40%)
BRCA	Train	UU,XX,A1,B6,PL,MS,A7,OK,LL,D8,E9,AC,AR,GI,UL,3C,LQ	17	386 (40%)
	Valid	E2,AN,AQ,4H,Z7,HN,5L,PE,WT,5T,W8,LD,S3,AO,JL	15	185 (19%)
	Test	A2,EW,GM,A8,BH,OL	6	394 (41%)
BLCA	Train	YC,GD,PQ,GV,E5,E7,YF,S5,FT,2F,BT,GU,C4,G2,FJ,MV,4Z,5N,SY,BL,LC,LT,H4	23	133 (39%)
	Valid	UY,ZF,K4,HQ	4	62 (18%)
	Test	DK,FD,GC,XF,CU	5	147 (43%)
PAAD	Train	HV,RB,2J,US,LB,HZ,OE,YB,M8	9	60 (40%)
	Valid	2L,FB,3A	3	30 (20%)
	Test	Q3,3E,F2,YY,S4,XN,Z5,H8,IB,PZ,XD,H6	12	60 (40%)
COAD	Train	3L,DM,AY,4N,G4,T9,CM,5M,A6,QG,QL,NH,4T,WS,AU,SS	16	146 (38%)
	Valid	D5,AZ,CK,F4	4	74 (19%)
	Test	AA,AD	2	169 (43%)
READ	Train	F5,EI,AF,EF,CL,AH,G5	7	58 (40%)
	Valid	CI,DY,BM,DC	4	17 (12%)
	Test	AG	1	71 (49%)
KIRC	Train	3Z,6D,B0,GK,CZ,G6,B8,EU,MM,AS,B4	11	185 (38%)
	Valid	A3,DV,AK,B2	4	95 (20%)
	Test	BP,CJ	2	206 (42%)
GBM	Train	41,81,06,OX	4	92 (40%)
	Valid	19,28,RR,4W	4	46 (20%)
	Test	12,08,15,27,87,14,26	7	92 (40%)

Table 4. Cross-institutional validation performance across multiple TCGA centers. The table shows C-index performance for different fusion methods across ten cancer types, with institutional separation maintained during data partitioning to ensure realistic generalization assessment. Standard deviations indicate performance consistency across contributing institutions.

Method	UCEC	LUAD	LGG	BRCA	BLCA	PAAD	COAD	READ	KIRC	GBM
**Three Modality Methods (Text, RNA, Image)**										
LiMOE	0.557±0.026	0.572±0.014	0.624±0.019	0.583±0.038	0.580±0.015	0.552±0.012	0.594±0.044	0.530±0.028	0.631±0.048	**0.574±0.013**
MAGGate	0.560±0.042	0.546±0.034	0.556±0.016	0.554±0.026	0.527±0.016	0.520±0.013	0.582±0.069	0.623±0.066	0.543±0.044	0.567±0.037
MuIT	0.532±0.019	0.581±0.029	0.557±0.037	0.545±0.040	0.535±0.017	**0.607±0.057**	0.581±0.025	0.610±0.057	0.539±0.015	0.553±0.022
TF	0.600±0.040	0.569±0.032	0.592±0.046	0.583±0.036	0.545±0.020	0.550±0.037	0.551±0.026	0.638±0.030	0.587±0.023	0.538±0.026
Cross Attn Fusion	0.608±0.035	0.578±0.043	0.622±0.023	0.593±0.075	0.530±0.011	0.519±0.018	0.553±0.016	0.661±0.097	0.678±0.018	0.566±0.007
Early Fusion	0.614±0.036	0.535±0.018	0.626±0.013	0.686±0.024	0.569±0.017	0.525±0.011	0.579±0.040	0.654±0.015	0.661±0.010	0.509±0.002
Late Fusion	0.539±0.016	0.532±0.018	0.552±0.033	0.544±0.019	0.521±0.019	0.534±0.028	0.591±0.015	0.557±0.017	0.516±0.017	0.544±0.020
**Two Modality Methods (Text, Image)**										
Cross Attn Fusion	0.623±0.033	0.526±0.015	0.623±0.036	0.616±0.064	0.546±0.025	0.528±0.022	0.593±0.135	0.635±0.152	0.633±0.075	0.519±0.015
Early Fusion	0.614±0.036	0.535±0.018	0.626±0.013	0.656±0.024	0.569±0.017	0.525±0.011	0.579±0.040	0.664±0.150	0.661±0.009	0.509±0.002
Late Fusion	0.558±0.019	0.527±0.011	0.562±0.031	0.540±0.021	0.519±0.015	0.556±0.040	0.591±0.015	0.557±0.017	0.516±0.014	0.544±0.020
**Ours**	**0.632±0.017**	**0.582±0.008**	**0.632±0.033**	**0.695±0.037**	**0.582±0.007**	0.578±0.034	**0.598±0.064**	**0.670±0.127**	**0.703±0.007**	0.554±0.034

Table 5. Supplementary Table S5. Statistical significance of performance superiority (paired t-test, Bonferroni corrected $\alpha = 0.0071$) and variance stability (F-test). Each cell format: mean C-index difference / variance reduction %. Significance: ** $p < 0.01$, * $p < 0.05$ (Bonferroni-corrected), †: baseline outperforms our method, -: not significant. Bold indicates statistically significant improvement with $p < \alpha$.

Cancer	LiMOE	MAGGate	MulT	TF	Cross Attn	Early Fus.	Late Fus.
UCEC	**+0.075****/57	**+0.072****/84	**+0.100****/20	+0.032/82	+0.024/76	+0.018/78	**+0.093****/-13
LUAD	+0.010/67	+0.036/94	+0.001/92	+0.013/94	+0.004/97	**+0.047***/80	**+0.050***/80
LGG	+0.008/-202	**+0.076****/-325	**+0.075****/20	**+0.040***/49	+0.010/-106	+0.006/-544	**+0.080****/0
BRCA	**+0.112****/5	**+0.141****/-103	**+0.150****/14	**+0.112****/-6	**+0.102****/76	+0.009/-138	**+0.151****/-279
BLCA	+0.002/78	**+0.055****/81	**+0.047****/83	**+0.037***/88	**+0.052****/60	+0.013/83	**+0.061****/86
PAAD	+0.026/-703	+0.058/-584	†**-0.029***/64	**+0.028***/16	**+0.059****/-257	+0.053/-855	**+0.044***/-47
COAD	+0.004/-112	**+0.016***/14	+0.017/-555	+0.047/-506	+0.045/> 10×	+0.019/-156	+0.007/> 10×
READ	+0.140/> 10×	+0.047/-270	+0.060/-396	+0.032/> 10×	+0.009/-71	+0.016/> 10×	+0.113/> 10×
KIRC	+0.072/98	**+0.160****/97	**+0.164****/78	**+0.116****/91	+0.025/85	**+0.042***/51	**+0.187****/83
GBM	†**-0.020***/-584	†-0.013/16	+0.001/-139	+0.016/-71	†-0.012/> 10×	+0.045/> 10×	+0.010/-189

Table 6. Supplementary Table S6. F-test analysis of variance stability between our method and baselines across 5 independent runs ($df_{base} = 4, df_{ours} = 4$). The F-statistic is defined as $F = \text{Var(Baseline)}/\text{Var(Ours)}$, where $F > 1$ denotes superior stability in our method. Significance levels: ** $p < 0.01$ ($F > 15.98$), * $p < 0.05$ ($F > 6.39$). Values below 1.0 indicate higher variance in our method, primarily observed in cohorts with high data sparsity.

Cancer	LiMOE	MAGGate	MulT	TF	Cross Attn	Early Fus.	Late Fus.
UCEC	2.34	6.10	1.25	5.54	4.24	4.48	0.89
LUAD	3.06	**18.06****	**13.14***	**16.00****	**28.89****	5.06	5.06
LGG	0.33	0.24	1.26	1.94	0.49	0.16	1.00
BRCA	1.05	0.49	1.17	0.95	4.11	0.42	0.26
BLCA	4.59	5.22	5.90	**8.16***	2.47	5.90	**7.37***
PAAD	0.12	0.15	2.81	1.18	0.28	0.10	0.68
COAD	0.47	1.16	0.15	0.17	0.06	0.39	0.05
READ	0.05	0.27	0.20	0.06	0.58	0.01	0.02
KIRC	**47.02****	**39.51****	4.59	**10.80***	**6.61***	2.04	5.90
GBM	0.15	1.18	0.42	0.58	0.04	0.01	0.35
